# Pharmacokinetics of doripenem in plasma and epithelial lining fluid (ELF): comparison of two dosage regimens

**DOI:** 10.1007/s00228-017-2327-y

**Published:** 2017-09-17

**Authors:** Zoe Oesterreicher, Iris Minichmayr, Robert Sauermann, Daniela Marhofer, Edith Lackner, Walter Jäger, Alexandra Maier-Salamon, Richard Schwameis, Charlotte Kloft, Markus Zeitlinger

**Affiliations:** 1 Department of Clinical Pharmacology, Medical University of Vienna, Vienna, Austria; 2 Department of Clinical Pharmacy and Biochemistry, Institute of Pharmacy, Freie Universitaet Berlin, Berlin, Germany; 3 Department of Anaesthesia and Intensive Care Medicine, Medical University of Vienna, Vienna, Austria; 4 Department of Clinical Pharmacy and Diagnostics, University of Vienna, Vienna, Austria

**Keywords:** Doripenem, Pharmacokinetics, Epithelial lining fluid, Intensive care, Pneumonia

## Abstract

**Purpose:**

In 2014, FDA released a warning for prescription of doripenem for ventilator-associated bacterial pneumonia due to unsatisfactory clinical cure rates. The present study explores if the observed lack of efficacy might be explained by insufficient target site pharmacokinetics in intensive care patients after two different infusion schemes.

**Methods:**

Plasma and bronchoalveolar lavage sampling was performed in 16 intubated patients with pneumonia receiving doripenem either as 1-h or as 4-h infusion. Doripenem concentrations were measured at steady state in plasma over 8 h, bronchoalvoelar lavage was performed in each patient once either after 0 h, 2 h, 4 h or 6 h.

**Results:**

In plasma, mean values of C_max_, T_max_ and AUC_0–8_ were 16.87 mg/L, 0.69 h and 52.98 mg/L^×^h after 1 h of infusion, and 12.94 mg/L, 3.21 h and 70.64 mg/L^×^h after 4 h of infusion, respectively. While the later t_max_ in plasma was with delay mirrored in the lung, for ELF, much lower concentrations were observed (C_max_, T_max_ and AUC_0–8_ after 1-h infusion of 4.6 mg/L, 2 h and 15.3 mg/L^×^h and after 4-h infusion 6.9 mg/L, 4 h and 14.8 mg/L^×^h).

**Conclusion:**

The difference in plasma pharmacokinetics after 1-h and 4-h infusion reflects in the concentration versus time profile in the lung, but concentration at the target site was not only considerably lower but also subject to high inter-individual variability. We hypothesise that insufficient concentrations at the target site might have contributed to the previously described lack of clinical efficacy and confirmed the demand for assessment of target site pharmacokinetics in larger patient collectives.

## Introduction

Sometimes, important lessons can be learned from failures. Doripenem is a carbapenem antibiotic with broad spectrum activity against Gram-positive and Gram-negative bacteria initially approved for the treatment of complicated intra-abdominal and urinary tract infections, as well as in Europe for the therapy of nosocomial including ventilator-associated pneumonia (VAP). In 2012, a large clinical trial comparing 7 days of treatment with 1 g of doripenem as a 4-h infusion every 8 h and 10 days of treatment with 1 g of imipenem/cilastatin as a 1-h infusion every 8 h showed increased risk of death and lower clinical cure rates when using doripenem compared with imipenem in patients with VAP [1]. Due to these results the FDA included a warning about the use of doripenem in patients with VAP in the drug label in 2014 [2]. In the same year, the marketing authorisation holder withdrew the European marketing authorisation of doripenem for all indications due to commercial reasons [3].

Antimicrobial efficacy depends on the concentrations of an antibiotic at the infection site, which may differ substantially from plasma concentrations [4–6]. Knowledge on plasma pharmacokinetics alone is therefore often not sufficient to allow for the appropriate estimation of bacterial killing in the targeted compartment [4, 7]. This single-centre descriptive pharmacokinetic study investigated doripenem concentrations in plasma and—representing the target site—in the epithelial lining fluid (ELF) of the lung with the aim to determine whether insufficient tissue concentrations at the target site might explain the observed lack of efficacy. Furthermore, two different dosing schemata of doripenem, i.e. standard infusion over 1 h versus extended infusion over 4 h were examined for the initially approved dosing regimen of 500 mg t.i.d to explore if longer infusion times lead to better serum and potentially also to target site target achievement.

## Methods

### Study subjects and treatment groups

Sixteen intubated intensive care patients who received doripenem for treatment of pneumonia were included in this study. Patients were split into two groups of which one received doripenem 500 mg over 1 h, the other one over 4 h. Inclusion criteria were age above 18 years, the administration of doripenem for therapeutical or prophylactic reasons for nosocomial or community-acquired pneumonia, as well as mechanical ventilation via endotracheal tube. Main exclusion criteria were a known allergy or hypersensitivity against the study drug, hemofiltration or haemodialysis or a ratio of the partial pressure of arterial oxygen to the fraction of inspired oxygen of less than 100 in combination with a positive end expiratory pressure of 20 cm of water or higher.

### Plasma sampling

Intensive plasma sampling over 8 h was performed for determination of antibiotic concentrations at steady state after (mean) 8.3 doses (range 3–24 doses prior to the study dose) in the 1-h infusion cohort and after 5.0 doses in the 4-h infusion cohort (range 3–9 doses).

### Bronchoalveolar lavage

Bronchoalveolar lavage (BAL) was performed once in each patient at one of the time points 0, 2, 4 or 6 h after start of infusion by using the pre-existing endotracheal tube for administration of three 20 mL aliquots of saline and subsequently collecting fluid after each aliquot. The fluid which was collected after the first aliquot was dismissed and not included into analysis. Concentrations in BAL samples were corrected using the urea method (volume of ELF (mL) = [total amount of urea in lavage fluid retrieved (mg)] / [concentration of urea in plasma (mg/mL)]) [8] in order to calculate concentrations in ELF.

### Analysis

The concentration of doripenem in plasma and ELF was determined by HPLC using a Dionex “UltiMate 3000” system (Dionex Corp., Sunnyvale, CA) with UV detection at 298 nm, chromatographic separation was carried out at 45 °C on a Hypersil BDS C18 column (5 μm, 250 × 4.6 mm I.D., Thermo Fisher Scientific, Inc., Waltham, MA), preceded by a Hypersil BDS C18 precolumn (5 μm, 10 × 4.6 mm I.D.). The mobile phase consisted of a continuous gradient mixed from ion-pair buffer, pH 3.0 (50 mM potassium phosphate with phosphoric acid and 5 mM heptanesulfonic acid (mobile phase A) and methanol (mobile phase B)). Calibration of the chromatogram was accomplished using the external standard method. Linear calibration curves were calculated from the peak areas of doripenem compared with the external standard by spiking drug-free human plasma and ELF with standard solutions of doripenem to obtain a concentration range of 0.01 to 10 μg/ml (average correlation coefficients > 0.99). The limit of quantification (LOQ) for doripenem in plasma and ultrafiltrate was 0.01 μg/ml. Coefficients of accuracy and precision for this compound were < 8.7%.

### Pharmacokinetic calculations

One ELF concentration of doripenem per patient and in total, two concentrations per time point per cohort were finally obtained, and mean values at the different time points were calculated. Pharmacokinetic analysis for determination of C_max_, T_max_, and AUC was performed using Kinetica version 3.0, InnaPhase Corporation. Since doripenem has negligible protein binding, below 10% of total plasma concentrations were used for calculations [9].

### Statistical calculation

Statistical calculation was performed using a commercially available programme IBM SPSS Statistics Version 20 (Armonk, NY, IBM Corp.). Correlation of creatinine clearance and AUC_0–8_ was calculated using Spearman correlation coefficient.

### Pharmacodynamic considerations

Since doripenem—like all beta-lactams—belongs to the class of antibiotics which exhibit time-depending killing, time above the MIC_50_ and MIC_90_ (T_>MIC_) of two main Gram-negative pathogens causing pneumonia were calculated for the generated data. MIC values used for calculations were chosen as found in the trial conducted by Kollef et al. [1] showing MIC_50_ of 1 mg/L and MIC_90_ of 128 mg/L for *Pseudomonas aeruginosa* and a MIC_50_ value of 2 mg/L and MIC_90_ value of 64 mg/L for *Acinetobacter baumanii.* Time over the MIC was directly determined by measurement of strongly enlarged figures. The fact that in case of severe infection, a PK/PD target of four times of the MIC value (*T*_>4xMIC_) was previously suggested to achieve optimal therapeutic effect, was included into PK/PD considerations [10].

## Results

### Study subjects

Demographic data and a selection of laboratory parameters are shown in Table 1.

**Table 1:** Characteristics (means ± standard deviation) of 16 study subjects

*n* = 16	Age year	Sex (male: female)	Weight kg	Creatinin clearance (mL/min)	LDH (U/L)	CRP (mg/dL)	Albumin (g/L)
1-h infusion	60 ± 13.7	7: 1	84.9 ± 16.3	112.4 ± 62	304.3 ± 99.6	14.3 ± 13.0	26.9 ± 4.4
4-h infusion	65.9 ± 8.9	5: 3	70.0 ± 21.3	76.2 ± 33.5	376.6 ± 240	9.6 ± 5.8	26.0 ± 3.4

### Pharmacokinetics

**Fig. 1:**
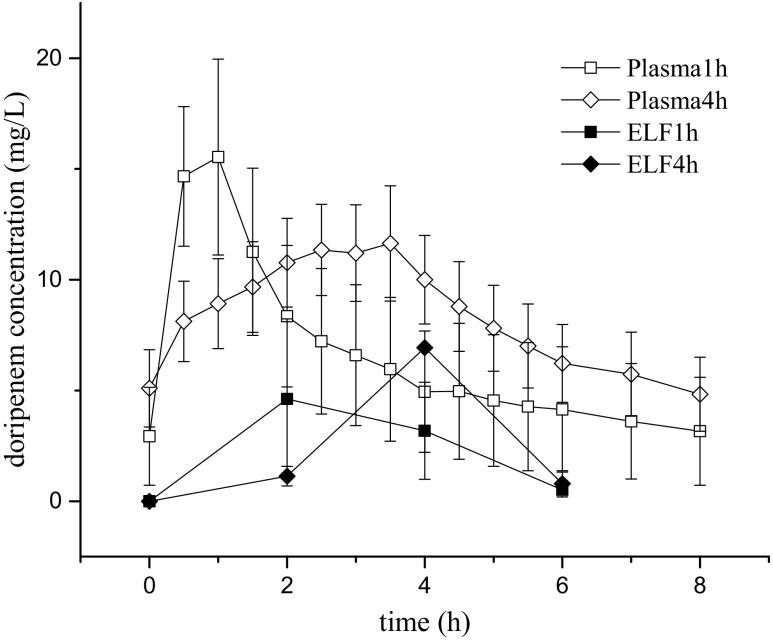
Mean (±SEM) concentration vs. time profiles of doripenem in plasma and in ELF obtained by BAL at 0, 2, 4, and 6 h after the start of doripenem infusion. Open symbols describe pharmacokinetics in plasma, closed symbols pharmacokinetics in ELF after an infusion duration of 1 h (squares) and 4 (diamonds) hours, respectively

**Fig. 2:**
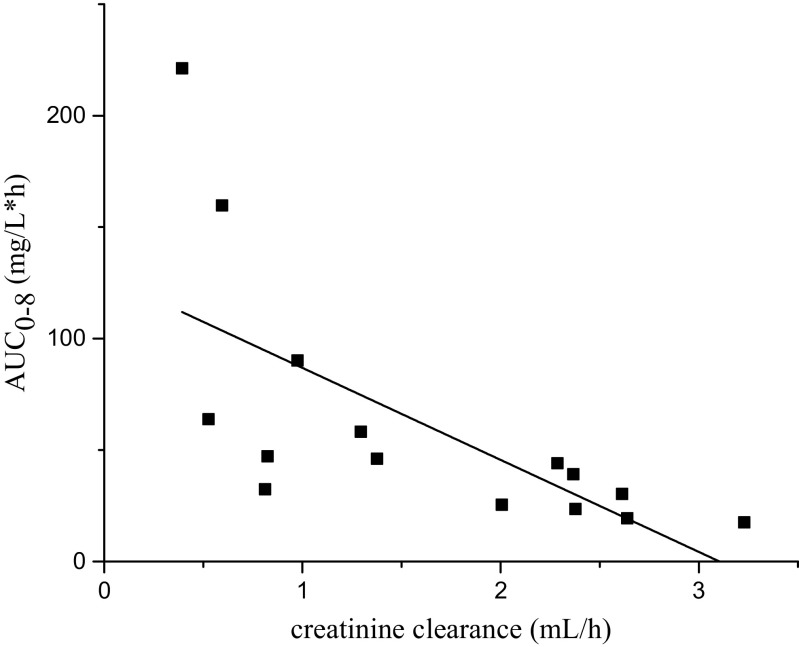
Correlation of creatinine clearance with AUC_0–8_ is significant at the 0.01 level (Spearman correlation coefficient 0.825)

In plasma, mean values of C_max_, T_max_ and AUC_0–8_ were 16.87 mg/L, 0.69 h and 52.98 mg/L^×^h after 1 h of infusion, and 12.94 mg/L, 3.21 h and 70.64 mg/L^×^h after 4 h of infusion, respectively. In ELF, C_max_, T_max_ and AUC_0–8_ after infusion over 1 h were 4.6 mg/L, 2 h and 15.3 mg/L^×^h and after infusion over 4 h 6.9 mg/L, 4 h and 14.8 mg/L^×^h, respectively. Pharmacokinetic data for plasma and ELF are shown in Table 2 and Fig. 1. A significant correlation at the 0.01 level was found between creatinine clearance and AUC_0–8_ in the present study as shown in Fig. 2.

**Table 2:** Pharmacokinetic parameters determined in plasma and ELF for standard and extended doripenem infusion (means ± standard error of the mean)

		C_max_ (μg/ml)	T_max_ (h)	AUC_0–8_ (μg/ml*h)	T/2 (h)	VD (L)	Cl (L/h)
Plasma	1 h	16.87 ± 4.10	0.69 ± 0.09	52.98 ± 24.29	2.93 ± 0.84	48.50 ± 12.76	15.71 ± 2.91
	4 h	12.94 ± 2.40	3.21 ± 0.24	70.64 ± 16.39	4.04 ± 0.83	33.61 ± 5.55	6.96 ± 1.41
ELF	1 h	4.6	2	15.3			
	4 h	6.9	4	14.8

### Pharmacodynamics

In plasma, the investigated dosing regimens achieved mean T_>MIC50_ values of 78 and 61% for the 1-h infusion and values of 100 and 98% for the 4-h infusion for *P. aeruginosa* and *A. baumanii*, respectively. However, employing the target of four times over the MIC *T*_>4xMIC_, the 1-h infusion led to 39 and 24% *T*_>4xMIC50_ for *P. aeruginosa and A. baumanii*, respectively, whereas the extended infusion scheme achieved values of 81 and 45% of *T*_>4xMIC50_. Neither infusion scheme reached at any time point of the MIC_90_ values of the two pathogens.

Investigating T_>MIC50_ in ELF, neither one of the infusion schemata could lead to doripenem concentrations above 1 mg/L (equivalent to MIC_50_ of *P. aeruginosa*) over the full dosing interval, though both dosing regimens could provoke concentrations above 1 mg/L at the 2-h as well as the 4-h time point.

## Discussion

We set out to investigate if insufficient concentrations at the infection site in the lung can explain the previous described failure of doripenem when treating VAP and whether longer infusion times can help to optimize target attainment both in plasma and at the target site. Due to the limited number of patient included and high variability of determined PK parameters, especially in the lung, these questions could only partially be answered. On the other hand, we were able to show that the observed variability intrinsically might be a factor that might have resulted in treatment failure in a relevant proportion of patients and that modification of the dosing regimen also reflects in target site PK.

In order to allow for a hypothesis explaining the insufficient efficacy of doripenem observed by Kollef et al. based on plasma PK, the following assumptions have to be made. First, as the actual MIC values of the pathogens that caused the VAP in that study were not available, only MIC_50_ levels could be used for PK/PD considerations. Second, individual PK profiles are not published for the study by Kollef et al., thus we could only estimate plasma exposure in that study by doubling plasma concentrations at every time point in all patients of the present study after the identical duration of infusion (4 h) to compensate for differences in dosing. Third, the fact that blood concentrations over four times the MIC might be needed in case of severe infections for beta-lactam antibiotics have to be considered [11]. Taking all these assumptions into account—including measuring time over MIC in figures after doubling plasma concentrations—the prolonged infusion regimen would have resulted in mean *T*_>4xMIC50_ of 81 and 45% for *P. aeruginosa* and *A. baumanii*, respectively. For beta-lactams, values between 40 and 60% T_>MIC_ have been associated with optimal killing in in vivo and in vitro PK/PD models by Craig et al., [12] therefore, for both strains time over MIC_50_ would be in or above the range of 40–60%,suggesting that good antimicrobial efficacy might be expected.

Still, also in patients suffering from VAP caused by *P. aeruginosa*, the clinical cure rate was numerically lower for patients with *P. aeruginosa* VAP in the doripenem arm compared to the imipenem-cilastatin arm (41.2 versus 60.0%). Therefore, one might speculate that the observed lack of efficacy might be due to insufficient target site penetration in the critically ill population rather than with subtherapeutic plasma levels. Although not enough data for thorough PK-PD calculations for the lung was generated in the present study, a difference between the ELF concentration-time profiles after the two investigated infusion schemes was observed. As shown in Fig. 1, differences in dosing regimens and thereby modified plasma PK indeed reflects in changes in target site pharmacokinetics in ELF. However, none of the two profiles seem to sufficiently cover concentrations above the thresholds of 4 or 8 mg/L (= 4× MIC_50_ of pathogens with MIC 1 mg/L or 2 mg/L) which might explain why potential benefits of a prolonged infusion scheme did not translate into improved clinical endpoints when treating VAP.

For reliable determination of the time above the MIC in ELF, more BAL measurements, either by including more patients or by repeated samples per patient, would be necessary to permit PK-PD calculations and to compensate for the high inter-individual variability, i.e. differences in concentrations up to the factor eight at a single-time-point. Different factors like organ dysfunction, septic shock, concomitant medication or capillary leakage have been attributed to this variability [4, 7, 13, 14]. However, more BAL time points per patient might be problematic from the ethical point of view and repeated BAL procedure in one patient might falsify data. Moreover, while including more patients in this study would allow for better target attainment analysis for the overall population it might not change the main outcome, i.e. that inter-individual variability has to be expected to be very high, and despite the fact that plasma concentrations are mirrored in ELF, target site concentrations in an individual patient currently cannot be predicted.

A significant correlation has been found between creatinine clearance and AUC_0–8_ in the present study as shown in Fig. 2. Thereby, differences in creatinine clearance (112.4 vs. 76.2 mL/min for 1 and 4-h infusion, respectively) might also have contributed to higher AUC_0–8_ values found for the 4-h infusion regimen. In contrast to plasma pharmacokinetics, which is highly impacted by creatinine clearance, factors that are more difficult to measure, e.g. local inflammation in the lung or atelectasis, might additionally impact the target site penetration [15, 16]. Intracellular concentrations have not been determined in the present study, because beta-lactam antibiotics are not active against intracellular pathogens and no accumulation in phagocytes has been described [17]. Comparison of our data with pharmacokinetics in plasma and ELF determined by Justo et al. [18] in healthy adults after doripenem 500 mg administration as extended infusion allows a cautious conclusion on the difference between healthy subjects and patients. Plasma concentrations were up to 1.8-fold higher in patients than in healthy subjects (8.79 and 4.89 mg/L at 4.5-h time point). Likewise, C_max_ in ELF of 6.93 mg/L (4-h time point) in patients was higher than in healthy volunteers 1.67 mg/dL (4-5 h time point), but most importantly variability was lower.

Our study thereby highlights the importance of determining infection site pharmacokinetics but most importantly the need for further exploration of factors impacting target site pharmacokinetics in the respective patient category as e.g. the individual lung penetration of antibiotics in patients with severe pneumonia.

In summary, our data suggest the potential benefit of prolonged infusion in terms of PK/PD indices in plasma in the investigated population of severely ill patients. While it was shown that differences in the concentration-time profile in plasma did transfer to the lung, the small sample size limits the information value of BAL data. We can only hypothesize that insufficient infection site concentrations might have contributed to a previously observed lack of efficacy. Nevertheless the study confirms the demand for assessment of target site concentrations of antibiotics as early and throughout antimicrobial drug development in order to avoid therapeutic failure despite plasma PK/PD targets are achieved.
